# On Rupture of the Uterus

**Published:** 1841-01-23

**Authors:** Joseph Warrington

**Affiliations:** Lecturer on Midwifery, &c. &c.


MEDICAL EXAMINER.

DEVOTED TO MEDICINE, SURGERY, AND THE COLLATERAL SCIENCES.

No. 4.] PHILADELPHIA, SATURDAY, JANUARY 23, 1841. [Vol. IV.

### ORIGINAL COMMUNICATIONS.

### ON RUPTURE OF THE UTERUS.

By Joseph Warrington, M. D., Lecturer on Midwifery, &c. &c.

By the terms rupture and laceration, according to Duparque, we should understand every solution of continuity resulting from the forced distention of a soft body, in opposition to, or contradistinction of the term *fracture*, which is especially applicable to an indirect division of bodies slightly or not at all distensible. Thus, in the animal economy, the soft tissues may be *ruptured* or *lacerated*, whilst the bony portions may be fractured or crushed.

When solution of continuity takes place in the body of a tissue by distention, it may be properly said to be *ruptured*. While, if solution of continuity commence in the free edges of membranous tissues, or parts which have a flattened form, *laceration* may be said to have occurred.

Thus, according to this gentleman, the body of the uterus may be *ruptured*, its neck may be *lacerated*, vertically.

The perinæum may be *ruptured* in its *centre*, while the fourchette or posterior commissure of the vulva may be *lacerated*.

When the accident takes place from mechanical injury, it is called *accidental rupture* or *laceration*;—but, when from irregular action of the uterus itself, it has received the designation of *spontaneous* rupture or laceration.

This last species of rupture from violent action of the organ itself may take place under two varieties of circumstances,—thus, first, when the pelvis is of a natural size, and the orifice of the uterus undilated, and in a state of extreme rigidity, and therefore undilatable by any safe exertion of the natural powers. Or it may happen from deformity of the pelvis, the membranes being ruptured, the waters evacuated, and the pains at the time violent.

Rupture of the uterus may, according to Davis, take place in any direction. When the injury is inflicted by the practitioner's hand, the rupture is for the most part found after death occupying the fundus, or the superior part of the body.

When it takes place spontaneously in consequence of inordinate resistance, from want of sufficient capacity of the pelvis, it usually implicates the portions of the uterus which are known commonly to correspond with the localities of the promontory of the sacrum behind, and of the linea ileo-pectinea at the sides and in front.

These ruptures most frequently take place transversely, and very rarely in longitudinal directions.

Blundell met with a case in which the rent was longitudinal, and in that part of one side of the uterus which is situated between the folds of the broad ligament.

Radford met with a case of longitudinal rupture from the neck to the base, (or fundus.)

The direction of the rupture probably depends upon the arrangement of the muscular fibres of the uterus: thus, when the force of the cause of the rupture is applied upon the cervix, the solution of continuity takes place in a transverse direction,—while, if it take place in the body, it is more likely to be longitudinal or oblique.

Blundell says, ruptures of the uterus are not of very uncommon occurrence: that they are not usually made the subject of conversation, because those who have the misfortune to occasion death in this manner, are naturally desirous of concealing the fact; yet he says frankly, from what he has seen himself, and from what he had learned from his obstetric friends, he was fully persuaded that lacerations of the womb are by no means unfrequent, and they require, therefore, our diligent study, both with regard to their prevention and cure.

Any causes which greatly distend the uterus, particularly those which put its fibres upon the stretch pathologically, may occasion rupture of this organ.

Thûs a case is recorded by a French writer of a female who had ceased to menstruate at 40 years. At 50 she perceived a tumor was forming in her abdomen—It increased gradually until its size became enormous. She was assured that it was formed in or by development of the uterus.

In one of the paroxysms of pain which became insupportable, she experienced a peculiar sensation in the abdomen. The pains then ceased, the tumor in the hypogastrium became flattened, the patient succumbed, and died the next day. At the autopsy the peritonæal cavity was found filled by an enormous amount of black and putrid blood; the uterus lay dilated and open, with its parieties consistent and thick, except about the fundus, where they were thinner, and at one portion presented an opening with ragged or lacerated edges. The cervix was cartilaginous and completely obliterated.

From these appearances and the signs which were manifested during life, it is evident that the effused blood escaped from the uterine cavity into the abdomen, through the rupture which took place at the fundus of the uterus.

It is, however, more particularly when the uterus is in a state of gravidity, that we may expect its rupture to occur.

Burns says, rupture of the gravid uterus may take place at any period of gestation. Thus according to him, the uterus sometimes, in the early months of gestation, is opened by a kind of ulcer, and occasionally by a species of slough, either of which states proceeds from previous disease in a part of the womb.

There may be pain attending this process, but in such instances as were known to Dr. Burns, there had been none. "The patient, without any evident cause, has been siezed with great sickness, and fits of fainting, which in a few hours have proved fatal."

Rupture of the uterus may be the consequence of mental agitation. Thus in a case under the care of Dr. Percival, the patient ascribed the rupture of her uterus to a fright; Dr. Underwood's, to mental agitation; Duparque's, to anger. The following case quoted at length from Professor Davis, of the London University, is highly interesting, as showing the effect of moral cause, and proving that although lacerations of the uterus usually make their way through the entire substance of its parietes; there are rare instances in which the loss of continuity has implicated the peritoneal tunic only.

"A poor woman, a patient of the Maternity Charity of London, had waited upon a committee of gentlemen, to make application for a contribution out of the funds which they were appointed to distribute; but the pecuniary assistance sought, for reasons not here necessary to state, was refused. This denial was instantly productive of a total change in her physical condition. From being able to have walked about half a mile to the place of meeting of the committee in question, she was suddenly bereft of all capacity for locomotion: and was not able to return home without the assistance of several friends, nor with that assistance until after the lapse of some hours. In the course of the same afternoon she was taken with labour pains. The labour soon became complicated with a slight sanguineous discharge.

About midnight she was seized with successive faintings, which induced the midwife to send for the assistance of Dr. Davis. He found the wretched patient in a very alarming state, exhibiting an appearance of much distress and and ghastliness, with short and difficult breathing, and the other characteristic symptoms, incident to rupture of the womb and its appendages. The uterus, however, was not totally incompetent for expellent efforts, and the head of the child was found being gradually pro pelled towards the outlet of the pelvis.

A dead child was born at four o'clock in the morning, the placenta was thrown off spontaneously, and shortly after withdrawn from the vagina by the gentlest traction.

No hæmorrhage followed, nor was there sustained throughout the whole process of the labour, the loss at most of twenty ounces of blood.

The patient gradually sunk after the labour was completed, and died in about three hours and a half subsequently.

At an early hour on the following day the abdomen was inspected. Some convolutions of the intestines were found floating in a great quantity of a dark, grumous, thickish fluid. On a first view it was considered probable that the uterus had sustained, as in ordinary cases of this nature, a rupture throughout its whole substance: but upon accurate examination of the actual injury, it was discovered that the source of this sanguineous discharge was a rupture of about two inches in length, together with several smaller fissures of the peritoneal covering of the posterior wall of the uterus, of which the deepest scarcely entered the twelfth of an inch into the subjacent parenchyma. During the moment of the first shock, which seemed to decide the fate of this poor woman, she felt, as she expressed herself, "as if struck with instant death."

Professor Davis asks, "was the child in this case, which was known to be alive in the early part of the morning, thrown into convulsive action, by the sudden moral shock which the mother sustained, and in consequence of the struggles of the fœtus, so produced, could the fatal injury have arisen?" The Professor concludes by saying, "that it might, is not, at all events, an improbable supposition."

Another case of rupture of the peritoneum, without implicating to any depth the parenchyma of the uterus, is said to have occurred in the practice of Sir Charles Clarke, and to be published in the first volume of the transactions of the Medico-Chirurgical Society.

Ramsbotham also furnishes the following case of rupture of the peritoneal covering of the uterus only, while the proper tissue of the organ remained entire. "A woman was delivered of her seventh child in the evening, after a painful labour; she became suddenly feeble and died the next morning. There was no external appearance of hæmorrhage. In the cavity of her abdomen was found a considerable quantity of blood, which had been poured out through a fissure several inches long in the peritoneal covering of the posterior face of the uterus; and apparently not involving the fleshy substance of the organ."

In the transactions for the improvement of medical and surgical knowledge, is given us a case of death from numerous lacerations of the peritoneal covering of the uterus.

"A woman 25 years old, was taken in labour with her first child at eight o'clock in the morning; every thing appeared to go on regularly for two hours. All at once, however, she was seized with pains in the abdomen, nausea, agitation, and great debility. She died at half past six in the evening, undelivered. The fœtus, however, was removed immediately afterward, and also the placenta.

At the autopsy no morbid appearance was found in the abdomen, all the viscera seeming to be sound. The uterus was slightly contracted; at its posterior surface about an ounce of blood was deposited in the duplication of the peritoneum. There were found on this posterior surface of the organ from forty to sixty transverse lacerations of the peritonæum."

Rupture of the uterus may take place from external violence, especially from powerful compression of the abdomen; as from blows, falls, &c.

In the Journal de Médecine for 1780, a case is detailed of a woman, seven months pregnant, who had her uterus ruptured by being forcibly squeezed between a wall and a carriage.

It was the opinion of Lamotte, Gregoire, Levret, Crantz, Astruc and some others, that the parietes of the uterus might be ruptured by the forcible movements of the fœtus while within its cavity.

In the Medical and Physical Journal for Nov. 1828, Dr. Else, a Surgeon of London, gives the following as a case of rupture of the uterus, supposed to be produced by the movement of the fœtus.

"Mrs. ——, aet. 20, experienced deep seated pains in the back and region of the uterus. A complication of symptoms seemed to threaten abortion. In returning from an excursion which she had made with her husband to Greenwich, she fainted, vomited, and died in less than an hour.

The uterus presented at its anterior portion a rupture five inches in length; it was vertical and extended to the placenta. The fœtus laying in front of the uterus was enveloped in its membranes and covered with coagulated blood, which was also effused into the pelvic cavity, and among the intestines. The uterus presented deep coloured spots or patches, at which the tissue was easily lacerable. The fœtus appeared to be healthy. Its movements in the uterus were the cause of its rupture."

I think myself it is very doubtful whether any motions of the fœtus, would be capable of producing such an accident, if there had not been a pathological condition of the uterus which had rendered this organ peculiarly easy of rupture. If it did occur in this case from the movements of the fœtus, the parietes of this uterus must have been exceedingly easy to be separated, for the death occurred at the time of quickening.

No doubt can exist but that the uterine tissue may undergo the process of ramollissement, but unfortunately we perhaps yet remain without sufficient evidence of its pre-existence to cause us to look with suspicious eye upon the subsequent parturient process, at which fatal consequences may ensue, unless we be prepared to aid in overcoming the resistance offered to the passage of the fœtus from the cavity of the uterus through the pelvic canal. Indeed, it sometimes happens that the fibres of the body, or fundus of the uterus yield to a fatal extent before the orifice of the uterus is dilated sufficiently to admit of any interference from art.

A case of this kind happened in the practice of one of my medical friends in this city, in which the patient had had slight uterine contractions for about thirty-six hours, gradually increasing in force for six hours longer, when they became very strong. Still, the os uteri was dilated only about enough to admit the extremities of two fingers; and although the contractions continued forcible, no further dilation occurred. She began to sink rapidly, all labour pains subsided, and she expired undelivered.

No other reason for the rupture in this case could be assigned by the physician in attendance than the probable softening of the uterine tissue, as she had been previously delivered in his presence of a child at term without any particular difficulty.

The resistances offered to the delivery of the child by defect in the pelvis from want of its amplitude in all its diameters, or, perhaps, more particularly by the shortening of one or more of its diameters in consequence of the projection of some osseous tumour or spicula of bone from some part of the pelvis, may, perhaps, be set down as amongst the common causes of rupture of the uterus. Of fatal accidents from this cause numerous cases are recorded. Thus, in a case which occurred to Dr. Dewees in 1796, there was a projection of bone thrown out from the left side of the symphysis pubes towards the base of the sacrum, so as to diminish its small diameter about half an inch. It presented a pointed extremity.

In a case which occurred in the practice of my friend Dr. Pierce, of this city, in 1835, the rupture was supposed to depend upon the presence of a spinous process upon the superior part of the symphysis pubis, about three-fourths of an inch long, and terminating in an apex.

In reference to the general diminution of the size of the pelvis, as a cause of rupture of the uterus, a case occurred in the practice of my friend and late obstetric pupil, Dr. Gee, in which, after as careful a measurement as could be made, the diameters of the superior strait were found to be half an inch shorter than usual, while the child was well developed.

In a case which occurred under my own notice in a patient who had been placed in the care of my pupil, Mr. Mitchell, of this city, who has reported the case at length in the Examiner, vol. iii., p. 616, in which, by the way, there are a few typographical errors, there is reason to believe, from the previous history of the case, that the pelvis was rather under size, and that the uterus was in a slightly pathological condition.

This, however, is a matter of inference rather than proof. She had already been the subject of three pregnancies,—the first, with a male child, was terminated by instruments—the two others, with female children, terminated spontaneously, but after very tedious and exhausting efforts. During gestation with her fourth, a large male child, she complained greatly of pain in the hypogastric region over the point at which the rupture took place.

If possible, it would be very desirable to the obstetric attendant to know what symptoms announce the state of the uterus which predisposes it to rupture, and particularly those which might apprize him that the accident is about to take place. It is acknowledged, by most accoucheurs of experience, that there are no diagnostic signs to be relied on—for all those which have been enumerated by some writers, have been known to occur in cases which have terminated favourably without any accident. Crantz has said, that "when a woman is threatened with rupture of the uterus, in a laborious labour, the belly is very prominent and tight, the vagina lengthened, and the orifice of the uterus very high—the pains are strong, leave little interval, and do not advance the delivery." To this narrative of premonitory symptoms, Levret has added, "that the pain the woman suffers, is always seated towards the middle of the epigastric region; that a last effort, or violent leap succeeds to the repeated struggles of the child, which announces its death, and the rupture of the uterus."

These occurrences are so very common in cases of laborious labour, that if they were always premonitory we should always be kept in a state of the most intense anxiety respecting the result; and perhaps sometimes prompted to the premature resort to measures, either manual or instrumental, with a view to hasten delivery, and thus parry off such terrible consequences.

Baudelocque says, rupture of the uterus has often taken place without being preceded by any of the symptoms just enumerated, and has not happened in other cases where their union seemed to declare it inevitable;—and further says, that if we were to take them for our guide, we should sometimes trench upon the rights of nature, by performing a delivery which she would have been able to terminate without inconvenience; and Dewees has added to this remark, that it would be extremely hazardous to act upon the presumption that a rupture of the uterus was about to take place, because of the symptoms referred to by Crantz and Levret; and he asks, who could justify the employment of the forceps, or crotchet, or preform the difficult and oftentimes dangerous operation of turning upon a mere surmise that this accident might take place?

Admitting, then, the great uncertainty of the signs enumerated as precursory of rupture, those which accompany or immediately succeed the accident, are much more to be depended upon.

Thus the woman feels, in most instances, an acute pain in the place at which the rent occurred,—she generally cries out, that something terrible has happened within her,—and it is said that at this moment an audible noise has been heard by some of the persons surrounding her—a discharge of blood, of greater or less extent, takes place from the vagina—her face becomes pale and cold—her respiration hurried, her stomach sick—vomiting takes place, sometimes of the common contents of the stomach only, at other times of very dark, even a black colored substance, resembling coffee grounds. The pulse becomes frequent, small, fluttering, or extinct. She complains of a mist before her eyes, loss of sight, and extreme feebleness or faintness; a cold clammy sweat bedews the whole surface of the body, and if not speedily relieved, convulsions and death follow.

These symptoms are, according to Dewees, modified by several circumstances.

1st. Whether it be the uterus itself, or its connexion with the vagina, that may be ruptured.

2d. Whether the child has escaped in part, or entirely into the cavity of the abdomen.

3d. Whether the lesion has passed through the substance of the uterus alone, or has penetrated the peritonæum.

For (1st.) When the rupture has taken place in either the body or neck of the uterus, the pains either cease, or slacken so much as not to propel the child, if it be still retained within the uterus.

(2d.) When the child escapes entirely into the cavity of the abdomen through the torn uterus, the most distressing and alarming symptoms quickly follow; if partially protruded, further contractions of the organ may effect the delivery of the child, or retain it in such a position as to favour its delivery by art.

(3d.) Should the wound stop at the peritoneal covering of the uterus, and not penetrate the abdomen, there is reason to believe that the symptoms will not only be milder, but the chance of recovery increased.

Although the train of symptoms indicating the occurrence of rupture in the uterus may be considered as having been generally given, in the group just quoted from Dr. Dewees, it should be born in mind, that there are instances of this unfortunate accident, in which other symptoms become prominent.

Thus, in a case detailed by Mr. Douglass, the head of the child was resting on the perinæum, when the lady, who had been subject to cramp, uttered a violent cry, and the head receded.

Mr. Goldson had a patient, who complained of cramp in the leg in the interval of the labour pains; and in the instant when the rupture happened, she exclaimed, "the cramp!"

In the case which occurred under my own notice, in the interval of severe expulsive contractions, the woman was supplied with a draught of cold water. Instantly a new contraction took place, and when it had endured a few moments, she suddenly let go her hold of the napkin by which she was supported, placed her hands on the epigastric region and exclaimed—" Oh! all the pain has gone up here!"

The pulse does not always become so considerably changed in force and frequency at the instant of the rupture, as some descriptions would dispose us to think.

Thus, in the case which happened to Dr. Gee, the pulse remained full and strong, even after the cadaverous vomiting ensued.

In the case of Mary M'Cutten, already alluded to, although the vomiting was not then very urgent: the pulse had so much firmness, as to induce me, under counsel, to have the woman bled from the arm, and that, too, at a time, as we have since believed, when blood was escaping into the cavity of the abdomen.

What is the most positive sign of rupture of the uterus ?

Probably that afforded by the introduction of the finger into the vagina, and the detection of the fact that the part which a short time before had presented in a certain manner, is now beyond reach.

This, though most strongly to be relied upon, does not always clear the diagnosis, for Denman says he knew two cases in which it appeared that the uterus was ruptured by the very effort which expelled the child. Blundell states, that in some rare cases the child is expelled, notwithstanding the rupture, it occurring probably from the very pain by which the birth is completed.

It is easy enough to imagine that when rupture has occurred in the latter part of labour, where there is a small or distorted pelvis, that the cranium of the fœtus may be so locked in the cavity of the bony canal, that it would not recede if both the tonic and alternate contractions of the uterus were completely to subside.

Neither the presence nor absence of hæmorrhage can afford any aid in diagnosticating this affection; for, although Dewees has enumerated amongst the symptoms of the occurrence of rupture, " a discharge of blood of greater or less extent," yet it may be clearly inferred from many cases on record, and from my own case and those which have been communicated to me, that no appreciable hæmorrhage takes place externally, even in cases in which the rupture is large enough to allow the child to escape into the cavity of the abdomen.

It is, therefore, to be inferred, that the positive signs of rupture are not always present, when this accident has occurred to some, indeed, to a fatal extent.

Blundell says that, " alarmed by the unexpected symptoms, viz. the symptoms of collapse already enumerated, you lay your hand upon the abdomen, and through the abdominal coverings you distinctly feel the child, and its different members, lying out of the womb, among the viscera."

This cannot always be relied upon; for the rupture is not always large enough to allow the instant escape of the child. Sometimes, in deed, the peritoneal coat, may remain entire, and thus prevent the escape of the fœtus from the uterine cavity.

In the case which occurred to me already mentioned, this diagnostic sign was very obscure.

What, then, is the positive sign, or what are the positive signs by which rupture of the uterus may be satisfactorily determined, during the life of the patient?

This is a subject which will require further investigation, and it is much to be wished that the histories of all cases of rupture of the uterus could be faithfully recorded, and the proper inferences drawn from them.

Suppose, however, we have this train of symptoms, and a suspension of the uterine contractions, with an apparently rapid ebbing of the vital powers? What is the accoucheur to do in such a dilemma?

It must be evident that if the suspension of pains and the occurrence of prostration arise from any other cause than rupture of the uterus, or a fatal lesion of some important organ, we ought rather to encourage the repose of the patient, or resort to the administration of some medicines calculated to enable the powers of the woman to rally. Would not prompt delivery here be contra-indicated?

On the other hand, could the professional attendant delay an instant the effort to delivery if he knew that a rupture of the uterus had occurred ?

So much, then, for the importance of accurate diagnosis, if it were possible to arrive at it.

We have said that circumstances might occur which might make this diagnosis difficult in some cases. I would not be understood to apply this difficulty to all cases; for when the fœtus does considerably recede from the upper strait, in which it had just before been engaged, and when the parietes of the abdomen are sufficiently thin to admit of feeling through them the angularities of the fœtus, when these could not have been felt before, the evidences of extensive and dangerous lesion of the uterus are sufficiently strong to justify prompt measures for delivery, with the view to rescue the child, and, *possibly*, also the mother.

When we have so far ascertained the nature of the accident, we become bound to use the appropriate means of delivery. These are, first, to attempt delivery through the natural passages; secondly, to open the cavity of the abdomen, and thence extract the fœtus. Most authors agree with Dewees that we may perform the first, whenever the neck, or its union with the vagina, is the seat of laceration, provided the pelvis be well formed, and the child has escaped into the cavity of the abdomen; the feet of the child should be sought for, and the delivery accomplished as in an ordinary case of turning,—but, should the pelvis be so contracted as not to permit the child's head to pass, then the operation of gastrotomy should be resorted to.

If but a portion of the child should have escaped through the rupture, and the head be still in the pelvis, the forceps should be used, or, if we are certain of the child's death, the cranium may be opened, its size lessened, and delivery completed by the crotchet.

Suppose the body, or fundus, or both, to be involved in the rupture, and the child has escaped through this part into the cavity of the abdomen, the delivery through the natural passages may be difficult or impossible, even when there is no defect of the pelvis; for the uterus will most probably contract itself so much as to render the re-passage of the child impracticable; and in this case the only chance of success for the safety of the child is the immediate resort to incision of the abdominal parietes,—and if there be any obvious deformity of the pelvis, no other course offers the least prospect of success.

Blundell, whose style of writing would seem to indicate that he never shrunk from professional duty, gives the following judicious counsel. "Let the mind in these dreadful emergencies be kept tranquil and unshaken: unless you are undisturbed and settled steadily upon obstetric principles, you are unfit to act. If you are unequal to the duty, give up the management of the case altogether, and send for further assistance. Do not mislead yourselves with a notion that these cases are desperate, and, therefore, that it matters little what is done by the patient. One recovery I have myself witnessed, and there are others on record."

The case of Blundell's is too interesting to be passed over without repetition.

"A woman in the neighborhood of Guy's hospital had a contracted pelvis; I was called on, in consequence of collapse of the strength, and when I examined I found the child laying in the peritoneal sac distinct from the uterus, the aperture of which was contracted, and I found further, a large transverse rent opposite to the bladder. Well, in this case, agreeably to the rule, I determined to turn; and for this purpose, introducing my hand into the peritoneal sac, I percieved the intestines, felt the beat of the large abdominal arteries, touched the edge of the liver, and ultimately reaching the feet of child, I withdrew it by the operation of turning, subsequently abstracting the placenta and membranes, the woman recovering in a few weeks afterwards.

About five years after the recovery, I saw her, not so vigorous as before the accident, but nevertheless tolerably well. On careful examination at this time the os uteri was found to present the natural characters, and not a vestige of the cicatrice was discoverable in the vagina any where, above, or below; the rupture, therefore, had been above, in the uterus itself. When in this case my hand was introduced to turn the fœtus, the uterus, large as a child's head, was felt lying upon the promontory of the sacrum, above and behind the rent."

